# Headspace Solid-Phase Microextraction Analysis of Volatile Components in *Narcissus tazetta* var. *chinensis* Roem

**DOI:** 10.3390/molecules181113723

**Published:** 2013-11-06

**Authors:** Hsin-Chun Chen, Hai-Shan Chi, Li-Yun Lin

**Affiliations:** 1 Department of Cosmeceutics, China Medical University, Taichung 404, Taiwan; 2 Department of Horticultural Science and Graduate Institute of Horticulture, National Chiayi University, Chiayi 600, Taiwan; E-Mail: hschi@mail.ncyu.edu.tw; 3 Department of Food Science and Technology, Hungkuang University, Taichung 433, Taiwan; E-Mail: lylin@sunrise.hk.edu.tw

**Keywords:** Chinese narcissus, *Narcissus tazetta* var. *chinensis* Roem, HS-SPME, volatile compound

## Abstract

The volatile components in single-flowered and double-flowered Chinese narcissus were identified by headspace-solid phase microextraction (HS-SPME) coupled with GC and GC/MS. Changes in aroma during the vase-life (days 0, 1, 2, 3, 4, 5 and 6) of two samples were also studied. A total of 35 compounds were identified, of which all were present in single-flowered and 26 in double-flowered samples. The main aroma components were (*E*)-β-ocimene, and benzyl acetate. Single-flowered narcissus have a higher percentage of benzyl acetate, while double-flowered narcissus have a higher percentage of 1,8-cineole. In vase-life, the total volatile component content peaked on day 2 for single-flowered and day 3 for the double-flowered narcissus. For both single-flowered and double-flowered narcissus flowers, the total content of volatile components had decreased significantly by day 4.

## 1. Introduction

Chinese narcissus (*Narcissus tazetta* var. *chinensis* Roem) is a member of the Amaryllidaceae family and the Narcissus genus. It is a monocot plant whose flowers develop at high temperatures and bloom at lower temperatures. Featured during the Chinese Spring Festival, Chinese narcissus is a traditional and well-known Chinese flower with high economic and ornamental value [1]. It is a popular ornamental flower worldwide, especially in China and Taiwan, and narcissuses are widely cultivated in southern China [2]. Narcissus has a strong fragrance [3], that is highly valued in the fragrance industry [4].

**Table 1:** Comparisons of major volatile compounds reported in *Narcissus* flowers in published papers.

Plant species/variety	Major compound	Methods of testing/extraction	Ref.
*Narcissus poeticus* L.	α-terpineol, menthyl-(*E*)-isoeugenol, methyl-(*E*)-isoeugenol, and benzyl benzoate	headspace	[3]
*N.* *trevithian* and *N. geranium*	Authors did not present any quantitative data on the individual compounds. However, they reported *N.* *trevithian* contains more phenolic compounds and fewer esters compared to *N.* *geranium*	high vacuum distillation	[4]
*N.* *tazetta var. chinensis*	benzyl alcohol, α-terpineol, γ-phenylpropyl alcohol, 1,8-cineole, benzyl acetate, and linalool	hydrodistillation	[5]
*N.* *tazetta* florepleno	benzyl acetate, methyl anthranilate, benzyl alcohol, and linalool	hydrodistillation	[6]
Narcissus, two cultivars	benzyl acetate, benzyl alcohol, linalool, and indole	hydrodistillation	[7]
Fresh narcissus flowers	benzyl alcohol , and α-terpineol	headspace	[8]
Narcissus flowers	(*E*)-β-ocimene and α-terpineol	Likens-Nickerson extraction	[9]
Narcissus flowers	benzyl acetate, (*E*)-β-ocimene, 3,4-dimethoxytoluene, and 3,5-dimethoxytoluene	headspace	[10]
Zhangzhou narcissus flowers	benzyl acetate, benzyl alcohol, indole, 3,7-dimethyl-1,6-octadien-3-ol, and ρ-mentha-1,8-dien-4-yl acetate	hydrodistillation	[11]
*N.* *tazetta* var. *chinensis*	benzyl acetate, linalool, and 1,8-cineole	hydrodistillation	[12]
*N.* *tazetta* and *N. tazetta* subsp. tazetta	γ-terpinene for *N.* *tazetta* γ-terpinene, linalool, and benzyl acetate for *N. tazetta* subsp. tazetta	lab-prepared absolutes	[13]
*N.* *taztta* var. *chinensis*	benzyl acetate and (*E*)-β-ocimene	headspace and simultaneous distillation	[14]
*N.* *tazetta* and *N. serotinus*	*trans*-ocimene for *N.* *tazetta* benzyl acetate for *N. serotinus*.	hydrodistillation	[15]
*N. poeticus* L.	cinnamy alcohol, methyl isoeugenol, isoeugenol, methyl eugenol, α-terpinol, and phenyl propyl alcohol	hexane and supercritical CO_2_ extraction	[16]
*N. pseudonarcissus* L.	(*E*)-β-ocimene	headspace	[17]

Several studies on the volatile compounds of *Narcissus spp.* have been performed. Table 1 summarizes plant species/variety, method of testing/extraction, major volatile compounds and reference number in the published papers. 

It was also recently confirmed that the use of headspace analysis provides a more natural profile that studies using hydrodistillation of plant volatiles [18]. SPME allows the sampling of the volatiles emitted by living plants in a fast and easy way [19], allowing the volatile compounds present in the headspace of odoriferous flowers in different flowering to be studied, including compounds that may not be detectable by conventional methods, such as solvent extractions, and hydrodistillation, but can be detected using HS-SPME [20].

Many previous studies have investigated the aroma of Chinese narcissus flowers, but few of them have discussed changes in narcissus flower aroma during the vase period. The aim of this work was to utilize HS-SPME method to investigate the volatile components and to understand the changes in these aromas during various stages of the vase period of fresh Chinese narcissus flowers.

## 2. Results and Discussion

### 2.1. Analysis of the Volatiles in Fresh Single- and Double-Flowered Chinese Narcissus Flowers

The volatile compounds in the narcissus flowers were analyzed by headspace solid-phase microextraction coupled with GC and with GC-MS. Table 2, Table 3 and Table 4 show the 35 components that were identified in single-flowered samples, including 13 monoterpenes, three terpene alcohols, one terpene aldehyde, four terpene esters, one terpene oxide, one aromatic aldehyde, one aromatic alcohol, four aromatic esters, one sesquiterpene, three aliphatic esters, two hydrocarbons, and one other compound. A total of 26 components were identified in double-flowered samples, including 13 monoterpenes, three terpene alcohols, one terpene oxide, one aromatic alcohol, three aromatic esters, two aliphatic esters, two hydrocarbons, and one other compound. The main constituents of the Chinese narcissus flowers were (*E*)-β-ocimene (62.73%–66.06%), benzyl acetate (11.65%–25.02%), (*Z*)-β-ocimene, 1,8-cineole, and linalool.

In a study by Sakai *et al.* [5], (*Z*)-β-ocimene was not detected. However, in a later study by Sakai [7], this component was detected. In a study by Surburg *et al.* [17], different headspace methods were used to analyze the aroma of *Narcissus pseudonarcissus* L. The main component was (*E*)-β-ocimene; a contribution of 75% was detected by the dynamic headspace method, while 30.1% was detected by the vacuum headspace method. In a study by Arai [14], the headspace method was used to analyze *Narcissus tazetta* var. *chinensis*, and a 51.19% contribution of (*E*)-β-ocimene was detected. In this study, we used HS-SPME for analysis and found 62.73 and 66.06% contributions of (*E*)-β-ocimene for the two samples used in this study. However, as shown in Table 1, no form of β-ocimene was reported as one of the major volatile components in distilled samples, therefore we postulate that heating during distillation decreased the content of this compound that could be detected. In addition, we found that single-flowered narcissus are rich in esters, including benzyl acetate, phenethyl acetate, isoamyl acetate, prenyl acetate, and 3-hexyl acetate. Among these ester components, the benzyl acetate content was the highest. Benzyl acetate was also a major component of narcissus flower aroma [3,4,5,6,7,10,11,12,13,14]. As shown in Table 1, benzyl acetate was reported as the first major volatile component in several studies, probably because of the low content of ocimene. In this study, the content of benzyl acetate was 25.02%, next to (*E*)-β-ocimene (67.02%). Volatile compound isolation methods by headspace or by distillation gave different data from this comparison. It was confirmed again that the use of headspace analysis provides a more natural profile than studies using hydrodistillation of plant volatiles [17]. 

In a study by Van Dort *et al.* [4] prenyl acetate was detected as a minor component. Regarding the terpene alcohols, linalool, and α-terpineol were detected. Among these compounds, linalool is the major contributor to the aroma [5,6,7,10,12,13,14]. α-Terpineol was reported as a major aromatic component of narcissus flowers in studies by Joulain [8], Loo and Richard [9], and Ehret *et al.* [3]. Indole is known as being very important in floral odors [4]. Although the indole content is low, its aroma threshold is low; thus, a pleasing and strong fragrance is emitted, even at low concentration. Fresh Chinese *Narcissus* flowers were picked and immediately analyzed and could therefore be considered as fresh at the time of SPME. As for aliphatic hydrocarbons, pentadecane was not detected by Sakai *et al.* [5] or Sakai [7], but this component was detected by Loo *et al.* [9]. The presence of *n*-alkanes, as a biomarker of fresh flowers, is ascribed to the *Narcissus tazetta* var. *chinensis* Roem flowers, and not contamination. Li *et al.* [20] and Shang *et al.* [21] also cited *n*-alkanes as a biomarker of living flowers for *Michelia alba* and *Syring oblate* flowers. In addition, sesquiterpene-type components are almost undetectable, which may be due to the poor absorption of sesquiterpene components by SPME [22,23]. To summarize, the main components of the floral scent of narcissus flowers were (*E*)-β-ocimene, benzyl acetate, linalool, and indole. Among these components, benzyl acetate and (*E*)-β-ocimene have floral aromas [24]. 

### 2.2. Volatile Compounds over the Vase-Life of Narcissus tazetta *var*. chinensis *Roem*

Table 3 shows the aroma constituents of *Narcissus tazetta* var. *chinensis* Roem at different flowering stages (flower buds, day 0; early flower blooming, day 1; flower blooming, day 2–3, late flower blooming, day 4; senescence, day 5–6 as analyzed by GC and GC/MS. All samples were placed in a plant tissue culture laboratory with a controllable environment, and the room temperature was set at 25 °C. The vase life was 6 days. Every morning, the SPME method was used to extract aroma for analysis. The GC method was used for analysis and to compare the peak area content. As shown in Table 2, a total of 35 volatile compounds were identified for the single-flowered samples, for which the volatile components content peaked on day 2, decreasing significantly by day 3, and being lowest at day 6. Among these main components, (*E*)-β-cimene, prenyl acetate, and benzyl acetate have the highest aroma content on day 2, with contents that decreased significantly thereafter. Among the monoterpene components, α-pinene, sabinene, β-myrcene and γ-terpinene have the highest content on day 0, decreasing with time thereafter. 6-Methyl-5-hepten-2-one was undetected on days 0-5, but it was detected in trace amounts on day 5 and peaked on day 6. The odor of 6-methyl-5-hepten-2-one is metallic and wet-rubber-like as described by Chen *et al.* [24]. The compound was a speculated off-odor of the *Narcissus tazetta* var. *chinensis* Roem. A total of 25 volatile compounds were identified for the double-flowered samples, and the content of their volatile components peaked on day 3 of the vase period. Among these major components, the aroma contents of (*Z*)-β-ocimene, (*E*)-β-ocimene, benzyl acetate, and linalool peaked on day 3, decreasing significantly by day 4. Oyama-Okubo *et al.* [25] analyzed of the major scent compounds in cut flowers of ‘Casa Blanca’ lilies reported that total emissions of scent compounds peaked on the third day and then decreased. 

**Table 2:** Volatile compounds of fresh flowers of *Narcissus tazetta* var. *chinensis* Roem.

Compound	RI ^x^	RI ^y^	Content (%) ^z^	
			Single-flowered	Double-flowered
Monoterpenes				
α-Pinene	936	941	0.10 ± 0.01	0.31 ± 0.05
Sabinene	973	967	0.01 ± 0.00	0.15 ± 0.05
β-Pinene	978	971	0.02 ± 0.01	0.12 ± 0.06
Myrcene	987	983	0.61 ± 0.05	1.22 ± 0.09
α-Phellandrene	1002	1002	<0.01	<0.01
δ-3-Carene	1010	1004	0.05 ± 0.02	0.40 ± 0.15
α-Terpinene	1013	1011	0.05 ± 0.01	0.08 ± 0.01
Limonene	1025	1030	<0.01	<0.01
(*Z*)-β-Ocimene	1029	1037	1.80 ± 0.49	4.64 ± 0.78
(*E*)-β-Ocimene	1041	1040	62.73 ± 6.04	66.06 ± 11.01
γ-Terpinene	1051	1051	0.42 ± 0.11	0.27 ± 0.08
α-Terpinolene	1082	1085	0.04 ± 0.01	0.12 ± 0.03
*allo*-Ocimene	1113	1116	1.21 ± 0.07	1.22 ± 0.48
Monoterpene alcohols				
Linalool	1086	1087	1.14 ± 0.59	1.71 ± 0.35
α-Terpineol	1176	1174	0.28 ± 0.02	0.06 ± 0.02
Myrtenol	1178	1176	0.04 ± 0.00	0.04 ± 0.01
Monoterpene aldehyde				
Citronellal	1129	1129	<0.01	
Monoterpene esters				
Neryl acetate	1342	1345	<0.01	
Geranyl acetate	1362	1362	<0.01	
Methyl cinnamate	1354	1373	0.10 ± 0.01	
Cinnamyl acetate	1420	1422	0.02 ± 0.01	
Monoterpene oxide				
1,8-Cineole	1025	1025	1.49 ± 0.50	4.05 ± 0.35
Aromatic aldehyde				
Benzaldehyde	964	964	<0.01	
Aromatic alcohol				
Benzyl alcohol	1006	1032	0.06 ± 0.01	0.01 ± 0.00
Aromatic esters				
Benzyl acetate	1134	1165	25.02 ± 5.66	11.65 ± 4.22
Phenethyl acetate	1230	1269	1.12 ± 0.16	0.38 ± 0.08
3-Phenylpropyl acetate	1335	1357	0.07 ± 0.03	0.51 ± 0.04
Benzyl benzoate	1730	1769	0.17 ± 0.02	
Sesquiterpene				
β-Caryophyllene	1431	1430	0.04 ± 0.02	
Aliphatic esters				
Isoamyl acetate	893	886	0.06 ± 0.01	0.03 ± 0.01
Prenyl acetate	979	906	1.26 ± 0.29	2.18 ± 0.38
3-Hexenyl acetate	985	985	0.13 ± 0.03	
Hydrocarbons				
Pentadecane	1500	1500	0.23 ± 0.09	0.02 ± 0.01
Eicosane	2000	1983	0.08 ± 0.03	0.06 ± 0.03
Other				
Indole	1257	1295	0.33 ± 0.11	0.26 ± 0.04

^x^ Literature Retention indices obtained from [26,27,28] and reference were checked for all compounds on DB-1 column; ^y^ Retention indices obtained using series of *n*-alkanes (C_5_-C_25_) on DB-1 column; ^z^ Values are means ± SD of six replicates.

**Table 3:** Volatile compounds on vase life of *Narcissus tazetta* var. *chinensis* Roem.

Compound	RI ^x^	Single-flowered (peak areas) ^y^								Double-flowered (peak areas)						
		0	1	2	3	4	5	6		0	1	2	3	4	5	6
Monoterpenes																
α-Pinene	941	8.99bc	4.82d	4.28de	2.01e					11.28bc	13.23b	17.30a	18.19 a	9.65bc	9.50bc	
Sabinene	967	1.30d	0.69e	0.10f						5.17a	1.82c	2.61b	1.70c			
β-Pinene	976	1.21c	0.84c	0.73c						3.54b	2.03c	9.48a	1.87c			
Myrcene	983	30.88d	22.61e	25.15d	8.87fg	1.81g				29.16d	46.25c	68.39b	107.22a	25.84d	14.64ef	13.34f
α-Phellandrene	1002			<0.10												
δ-3-Carene	1004	2.43f	1.91f	1.90f	1.32f	1.11f				10.19e	20.35b	36.72a	21.80b	18.30c	17.10c	13.30d
α-Terpinene	1011	1.00cde	1.99b	2.74a						1.23cd	0.91e	0.89e	1.26c	1.27c	0.98de	0.42f
Limonene	1030			<0.10												
(*Z*)-β-ocimene	1037	43.81de	139.76c	144.44c	64.98d	9.12fg	2.81g	1.83g		28.70efg	137.02c	221.85b	698.81a	237.87b	72.20d	35.13ef
(*E*)-β-ocimene	1040	792.65ef	2271.78d	2299.77d	645.21f	27.79h	4.64h	2.24h		311.77g	2694.95c	3147.59b	9914.71a	3371.10b	1024.45e	659.79f
γ-Terpinene	1051	18.09c	9.50d	7.47d	4.20e	3.89e	1.48e	1.39e		8.72d	16.68c	18.88bc	21.77ab	22.62a	21.67ab	18.75c
α-Terpinolene	1085	2.41e	2.01e	1.76f						2.24e	3.23d	4.33c	8.31a	5.83b		
Allo-ocimene	1116	37.71f	44.83e	49.57d	15.48h	1.28j				7.87i	48.03de	58.36c	184.16a	70.96b	26.29g	16.65h
Monoterpene alcohols																
Linalool	1087	58.85g	112.42f	109.84f	51.48g	20.99i	17.78i	1.21j		21.43i	147.11de	188.91c	280.72a	243.05b	159.81d	32.88h
α-Terpineol	1174	12.11b	15.21a	3.61c	3.06c											
Myrtenol	1176	1.34g	1.80d	2.03b	1.45f						1.62e	1.95bc	3.48a	1.90c		
Monoterpene ketone																
6-Methyl-5-hepten-2-one	965						0.10b	11.10a								
Monoterpene esters																
Geranyl acetate	1362		4.49a	3.01b	2.10c											
Methyl cinnamate	1373		3.70e	3.76de	4.71cd	4.43cde	5.23c				4.22de	3.72de	11.06a	9.56b		
Cinnamyl acetate	1422						3.20c			6.08a	4.86b					
Monoterpene oxide																
1,8-Cineole	1025	143.23d	158.73d	144.65d	64.14g	11.56h	2.76h	1.45h		85.13f	222.04c	268.56b	450.91a	271.20b	122.62e	65.85fg
Aromatic alcohol																
Benzyl alcohol	1032	1.40b	1.82a	1.51b								0.72d	1.12c			
Aromatic esters																
Benzyl acetate	1165	675.59e	1324.56b	1390.43b	119.50hi	20.52j	7.10j	4.47j		186.44gh	927.25d	1023.59c	1598.03a	568.64f	256.70g	99.44i
Phenethyl acetate	1169	15.59g	24.91e	18.12fg	6.90h	2.78i	1.78i			46.14b	32.15d	20.86f	53.12a	38.77c	14.91g	7.81h
3-Phenylpropyl acetate	1357										6.43d	12.02c	63.45a	34.34b	6.37d	
Benzyl benzoate	1769				5.31a	3.79b	1.79c									
Sesquiterpene																
β-caryophyllene	1430		8.15a	2.56c	2.50c	4.32b	4.22b									
Aliphatic esters																
Isoamyl acetate	886	2.41g	2.94g	3.10g	11.15bc	8.13d	11.98b	6.10e		3.69g	4.15fg	5.80ef	5.85ef	9.64cd	17.54a	17.59a
Prenyl acetate	906	50.71e	62.61d	65.91d	40.82f	3.27h	2.05h			24.15g	108.26c	147.73a	125.11b	123.04b	44.73f	21.61g
3-Hexenyl acetate	985					1.03b	2.79a									
Hydrocarbons																
Pentadecane	1500	6.80e	11.61de	15.22d	18.73d	15.70d	15.00d	5.51e		50.10a	43.63abc	37.21c	47.12ab	42.67bc		
Eicosane	1983	7.87b	4.31d	3.49e	2.84f						5.03c	4.02d	11.61a	5.11c	2.08g	
Other																
Indole	1295	12.50ef	36.07b	34.48b	18.03a	1.81h	0.61h				30.26c	41.90a	19.40d	14.22e	10.52fg	9.35g

^x^ Retention indices, using paraffin (C_5_-C_25_) as references; ^y^ Values are means of six replicates. Values having different superscripts are significantly (*p* < 0.05) different.

**Table 4:** Peak areas of chemical groups of volatile compounds on vase life of *Narcissus tazetta* var. *chinensis* Roem.

Compounds	Single-flowered (peak areas)								Double-flowered (peak areas)						
	0	1	2	3	4	5	6		0	1	2	3	4	5	6
Monoterpenes	940.48	2500.74	2537.91	742.07	45.00	8.93	5.46		419.87	2984.5	3586.4	10979.8	3763.44	1186.83	757.38
Monoterpene alcohols	72.30	129.43	115.48	55.99	20.99	17.78	1.21		21.43	148.73	190.86	284.2	244.95	159.81	32.88
Monoterpene ketone						0.10	11.10								
Monoterpene esters		8.19	6.77	6.81	4.43	8.43			6.08	9.08	3.72	11.06	9.56		
Monoterpene oxide	143.23	158.73	144.65	64.14	11.56	2.76	1.45		85.13	222.04	268.56	450.91	271.2	122.62	65.85
Aromatic alcohols	1.40	1.82	1.51								0.72	1.12			
Aromatic esters	691.18	1349.47	1408.55	131.71	27.09	10.67	4.47		232.58	965.83	1056.47	1714.60	641.75	277.98	107.25
Sesquiterpene		8.15	2.56	2.50	4.32	4.22									
Aliphatic esters	53.12	65.55	69.01	51.97	12.43	16.82	6.10		27.84	112.41	153.53	130.96	132.68	62.27	39.20
Hydrocarbons	14.67	15.92	18.71	21.57	15.70	15.00	5.51		50.10	48.66	41.23	58.73	47.78	2.08	
Other	12.50	36.07	34.48	18.03	1.81	0.61				30.26	41.90	19.40	14.22	10.52	9.35
Totals (peak areas)	1928.88	4274.07	4339.63	1094.79	143.33	85.32	35.30		843.03	4521.51	5343.39	13650.78	5125.58	1822.11	1011.91

Notes: All the definitions of the symbols used in Table 3 mean values were also used in Table 4.

For both single-flowered and double-flowered narcissus flowers, the total content of volatile components had decreased significantly by day 4, and the total volatile component was lowest on day 6.

### 2.3. Comparison of Volatile Compounds from Single-Flowered and Double-Flowered of Narcissus tazetta *var*. chinensis *Roem*

Due to their different relative contents, the two narcissus flower types might have different scents. More ester compounds were identified in single-flowered samples than in double-flowered samples, including benzyl benzoate, and 3-hexenyl acetate, which may contribute to the aroma profile of the flowers. As shown in Table 4, Single-flowered samples have lower total peak areas and higher percentages of sesquiterpenes than the double-flowered samples. 

## 3. Experimental

### 3.1. Plant Materials

Single-flowered and double-flowered narcissus bulbs were purchased from Zhangzhou, Fujian Province, China. These bulbs were placed in pots containing water and then left under outdoor light. The bulbs were incubated for 35 days until they blossomed.

### 3.2. Methods

#### 3.2.1. Volatile Components of Narcissus Flowers

(1) Aroma components of the single- and double-flowered Chinese narcissus flowers: Fresh single- and double-flowered narcissus flowers in full bloom (ten each) were picked and immediately placed into sealed bottle. The SPME method was used to extract the aroma components. This experiment and all other experiments in this study were performed with six replicates.

(2) Volatile compounds during the vase-life of *Narcissus tazetta* var. *chinensis* Roem: Samples of narcissus bulbs about to flower were placed in a tissue culture laboratory controlled at 25 °C. The samples were exposed to 12 h of light and 12 h of darkness every day. Fresh budding single-flowered and double-flowered narcissus flowers (one flower each) were selected on a day then defined as day 0. These two samples were cut and inserted into two water-containing test bottle (precleaned # 27343 22-mL clear screw cap vials; Supelco, Bellefonte, PA, USA). From day 0 to day 6 at 10:00–12:00 in the morning, the SPME method was used to extract aroma to monitor the changes thereof.

#### 3.2.2. Analysis of Volatile Compounds

(1) HS-SPME analysis. A 50/30-μm divinylbenzene/carboxen/polydimethylsiloxane fiber (Supelco, Inc.) was used for aroma extraction. The SPME fiber was exposed to each sample for 30 min at 25 °C, after which each sample was injected into a gas chromatograph injection unit. Peak area data reported from the integrator was used for quantification. 

(2) Analysis of volatile components of samples by GC: Qualitative and quantitative analyses of the volatile compounds were conducted using an Agilent 6890 GC equipped with a 60 m × 0.25 mm i.d. DB-1 fused-silica capillary column with a film thickness of 0.25 μm and a flame ionization detector. The injector and detector temperatures were maintained at 250 °C and 300 °C, respectively. The oven temperature was held at 40 °C for 1 min and then raised to 200 °C at 2 °C/min and held for 9 min. The carrier gas (nitrogen) flow rate was 1 mL/min. Kovats indices were calculated for the separated components relative to a C_5_-C_25_ n-alkanes mixture [29]. 

(3) Analysis of volatile components of samples by GC-MS: The volatile compounds were identified using an Agilent 6890 GC equipped with a 60 m × 0.25 mm i.d. DB-1 fused-silica capillary column with a film thickness of 0.25 μm coupled to an Agilent model 5973 N MSD mass spectrometer (MS). The injector temperature was maintained at 250 °C. The GC conditions in the GC-MS analysis were the same as in the GC analysis. The carrier gas (helium) flow rate was 1 mL/min. The electron energy was 70 eV at 230 °C. The constituents were identified by matching their spectra with those recorded in a MS library (Wiley 7n). In addition, the constituents were confirmed by using the Kovats indices or GC retention time data with those of authentic standards or by publication literature. 

(4) Statistical Analysis: The data were subjected to a mono-factorial variance analysis, with Duncan’s multiple range method used by a significance of differences of *p* < 0.05 (SPSS Base 12.0).

## 4. Conclusions

Thirty-five volatile components were identified in Narcissus flowers. The main aroma components for narcissus flowers were (*Z*)-ocimene, benzyl acetate, linalool, and indole. More ester compounds were identified in single-flowered samples than in double-flowered samples, which may contribute to the aroma profile of the flowers. The double-flowered samples had higher contents of 1,8-cineole than the single-flowered samples. During the vase life, it was found that the total volatile content peak area was greatest for single-flowered samples on day 2, while that for double-flowered samples occurred on day 3. The volatile constituents throughout the vase life of *Narcissus tazetta* var. *chinensis* Roem were reported for the first time in this study.
